# Conservation of S20 as an Ineffective and Disposable IFNγ-Inducing Determinant of *Plasmodium* Sporozoites Indicates Diversion of Cellular Immunity

**DOI:** 10.3389/fmicb.2021.703804

**Published:** 2021-08-06

**Authors:** Calvin Hon, Johannes Friesen, Alyssa Ingmundson, Diana Scheppan, Julius C. R. Hafalla, Katja Müller, Kai Matuschewski

**Affiliations:** ^1^Department of Molecular Parasitology, Institute of Biology, Humboldt University, Berlin, Germany; ^2^Parasitology Unit, Max Planck Institute for Infection Biology, Berlin, Germany; ^3^Medical Care Unit Labor 28 GmbH, Berlin, Germany; ^4^Department of Infection Biology, London School of Hygiene & Tropical Medicine, London, United Kingdom

**Keywords:** malaria, *Plasmodium*, sporozoite, whole organism vaccine, antigen, CD8^+^ T cells, immunization, pre-erythrocytic stage

## Abstract

Despite many decades of research to develop a malaria vaccine, only one vaccine candidate has been explored in pivotal phase III clinical trials. This candidate subunit vaccine consists of a portion of a single *Plasmodium* antigen, circumsporozoite protein (CSP). This antigen was initially identified in the murine malaria model and shown to contain an immunodominant and protective CD8^+^ T cell epitope specific to the H-2K^*d*^ (BALB/c)-restricted genetic background. A high-content screen for CD8^+^ epitopes in the H2K^*b*^/D^*b*^ (C57BL/6)-restricted genetic background, identified two distinct dominant epitopes. In this study, we present a characterization of one corresponding antigen, the *Plasmodium* sporozoite-specific protein *S20*. *Plasmodium berghei S20* knockout sporozoites and liver stages developed normally *in vitro* and *in vivo*. This potent infectivity of *s20*(-) sporozoites permitted comparative analysis of knockout and wild-type parasites in cell-based vaccination. Protective immunity of irradiation-arrested *s20*(-) sporozoites in single, double and triple immunizations was similar to irradiated unaltered sporozoites in homologous challenge experiments. These findings demonstrate the presence of an immunogenic *Plasmodium* pre-erythrocytic determinant, which is not essential for eliciting protection. Although *S20* is not needed for colonization of the mammalian host and for initiation of a blood infection, it is conserved amongst *Plasmodium* species. Malarial parasites express conserved, immunogenic proteins that are not required to establish infection but might play potential roles in diverting cellular immune responses.

## Introduction

Sustained anti-malaria therapy, exposure prophylaxis, and vector control have resulted in stable incidence and mortality rates in the tropics (World Health Organisation, 2020). To reduce global malaria burden and transmission of *Plasmodium* parasites, access to a safe and efficacious prophylactic vaccine will be needed. An established method to achieve lasting sterile protection against natural *Plasmodium* sporozoite infection is intravenous administration of multiple doses of live γ-irradiation-attenuated sporozoites (γspz) (Nussenzweig et al., 1967; Clyde et al., 1973; Hoffman et al., 2002; Richie et al., 2015). Identification of immunogenic antigens and immune correlates of protection vs. sporozoite exposure remain research priorities to develop second-generation sporozoite vaccines.

Protective sporozoite-induced immunity is achieved by complementary humoral and cellular memory responses (Rodrigues et al., 1993; Hafalla et al., 2011). Previous studies using γspz vaccination in mice have revealed the pivotal role of circulating and liver-resident CD8^+^ T cells in mediating protective immunity to the pre-erythrocytic stages of the parasite by cytolytic killing (Ferreira et al., 1986; Schofield et al., 1987; Weiss et al., 1988; Guebre-Xabier et al., 1999; Krzych et al., 2000; Schmidt et al., 2008). Antigen-specific, cytolytic CD8^+^ T cells target infected hepatocytes, which present MHC I-restricted parasite peptides on the cell surface (Romero et al., 1989; Rodrigues et al., 1991). Upon peptide recognition by CD8^+^ T cells, cytokines such as interferon-γ (IFNγ) (Schofield et al., 1987) and tumor necrosis factor (TNF) (Butler et al., 2010; Depinay et al., 2011) are released along with pro-apoptotic pore-forming lytic proteins, perforin and granzymes. The central role of CD8^+^ T cells in pre-erythrocytic stage immunity has been further highlighted by several studies, where abrogation of immunity in γspz-immunized mice and non-human primates was observed after *in vivo* depletion of CD8^+^ T cells (Schofield et al., 1987; Weiss et al., 1988; Doolan and Hoffman, 2000; Weiss and Jiang, 2012). CD8^+^ T cells, however, are less likely to confer protection during the asexual parasite growth inside erythrocytes, simply because MHC I molecules are not found on the surface of mammalian erythrocytes. Accordingly, the key role of cytolytic CD8^+^ T cells in sustained protection is largely restricted to the first obligate parasite expansion phase in the liver.

Despite decades of considerable investments in malaria research, only one malaria vaccine has been licensed to-date, known as the RTS,S/AS01 (RTS,S Clinical Trials Partnership, 2015; Neafsey et al., 2015). This subunit vaccine is based on the major *Plasmodium falciparum* sporozoite surface antigen, termed circumsporozoite protein (CSP) (Cohen et al., 2010). CSP is highly conserved amongst *Plasmodium* species, and essential for sporozoite formation, motility and hepatocyte adhesion. It contains a central repeat structure, which likely further contributes to immune-dominance over minor sporozoite surface antigens (Zavala et al., 1983; Zavala et al., 1985). Modest protection offered by this vaccine is strongly associated with humoral immunity and, to a lesser extent, T-cell mediated responses (Stoute et al., 1998; Cohen et al., 2010). Employing murine malaria models it was shown that CSP contains immunogenic targets of protective H2-K^*d*^-restricted CD8^+^ T cell responses that are vital for protection against sporozoite infections (Rodrigues et al., 1991; Sedegah et al., 1992). But more recent studies have shown that sterile protection can also be achieved in the absence of CSP-specific T cells (Kumar et al., 2006; Grüner et al., 2007; Gibbins et al., 2020). While these findings might partly explain the inadequate protective efficacy of the RTS,S/AS01 vaccine they also highlight the need to investigate non-CSP antigens.

For the identification and evaluation of immunogenic pre-erythrocytic stage CD8^+^ T cell epitopes two inbred mice strains, H2-K/D^*b*^- and H2-K^*d*^-restricted C57BL/6 (B6) and BALB/c mice, respectively, have been largely used. Immunizing either mouse strains with *Plasmodium berghei* (*Pb*) or *Plasmodium yoelii* (*Py*) γspz is able to induce highly protective responses (Romero et al., 1989; Rodrigues et al., 1991; Sano et al., 2001). Candidate CD8^+^ T cell epitopes are typically selected based on the amount of IFNγ from activated CD8^+^ T cells upon stimulation with soluble peptides as a proxy for recognition of immunogenic MHC I-restricted epitopes. Between the two mouse strains, B6 mice appear to represent a more accurate model for immunological studies as the elicited H2-K^*b*^-restricted CD8^+^ T cell responses during sporozoite infection are more diverse. Reliance on multiple doses for protective immunity suggest a closer resemblance to that of humans, in contrast to the ease of eliciting protective immunity in BALB/c mice due to the immunodominance of the CSP epitope (Hafalla et al., 2013). Furthermore, the inability of B6 mice to recognize the H2-K^*d*^-restricted CSP epitope as a consequence of MHC haplotype restriction allows identification of non-CSP-mediated immunity. A genome-wide screening for candidate pre-erythrocytic stage H2-K/D^*b*^-restricted CD8^+^ T cell epitopes in B6 mice returned only two epitopes that correlate with protracted CD8^+^ T cell responses (Hafalla et al., 2013). One H2-D^*b*^-restricted epitope originated from the thrombospondin-related adhesion protein (TRAP_130__–__138_) and displayed cytotoxic CD8^+^ T cell responses after a single immunization with γspz. Importantly, partial protection could be achieved by eliciting high levels of TRAP-specific CD8^+^ T cells *via* a heterologous prime-boost regimen.

Strikingly, the other identified H2-K^*b*^-restricted target of CD8^+^ T cells (S20_318__–__326_) found in that screening, which maps to the sporozoite-specific gene 20 (Kaiser et al., 2004), did not induce cytotoxic immune responses. In good agreement, tolerization with S20_318__–__32__6_ peptide failed to completely reverse protection, in contrast to TRAP_130__–__138_ (Hafalla et al., 2013). In this study, we aimed to investigate this unusual CD8^+^ T cell response by generating *S20* knock-out parasites to confirm the origin of the epitope and to study potential contributions of S20_318__–__326_ toward protection in whole sporozoite immunizations.

## Results

### *S20* Is Highly Conserved Among *Plasmodium* Species and Primarily Expressed in Sporozoites

**FIGURE 1:**
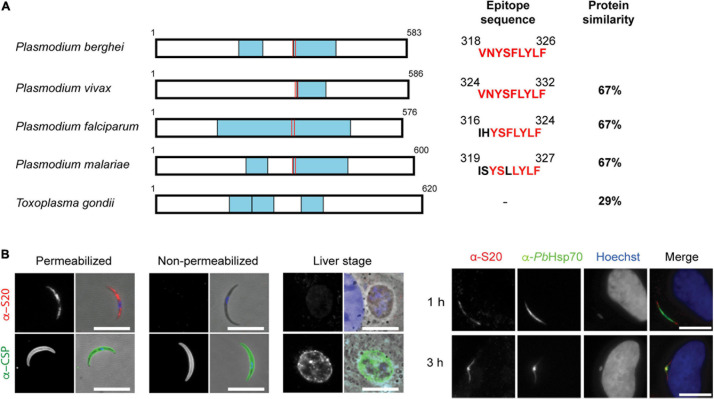
Conservation and expression of *Plasmodium berghei* S20. **(A)** Primary structure of *Plasmodium* and *Toxoplasma* S20 proteins. Predicted Kelch domains are boxed in blue, and the position of the epitope is shown by red lines. *P. berghei* (PBANKA_1429200; XP_034424116.1), *Plasmodium vivax* (PVP01_1432200; XP_001617233.1), *P. falciparum* (PF3D7_1213400; XP_001350539.1), *Plasmodium malariae* (PmUG01_14048200; XP_028864377.1), and *Toxoplasma gondii* (TGGT1_229000; EPR64070). Accession numbers are according to EuPathDB (Aurrecoechea et al., 2017), and alignments were generated using the BLAST algorithm (Altschul et al., 1990). **(B)** Detection of *P. berghei S20* by indirect immunofluorescence. Shown are immune stainings of permeabilized and non-permeabilized sporozoites, early liver stages 24 h after infection, and infected hepatoma cells 1 and 3 h after sporozoite addition. Stainings were done with an anti-*Pb*S20 serum, anti-*Pb*CSP antibody, anti-*Pb*HSP70 antibody and corresponding secondary antibodies. Shown are immune stainings and merge, including DNA stains (Hoechst, blue) and bright field images. Scale bars, 10 μm.

Since the sporozoite-specific protein S20 (PBANKA_1429200) was first identified in *Vinckeia* species (Kaiser et al., 2004), we first verified whether it is also present in other *Plasmodium* species and *Toxoplasma gondii*. This analysis revealed S20 orthologs in all *Plasmodium* species and a similar protein in *T. gondii* (TGGT1_229000). Examples of representative apicomplexan S20 orthologs are displayed in Figure 1A. S20 orthologs are also present in other Coccidia, for instance *Sarcocystis neurona* (SRCN_6348), *Besnoitia besnoiti* (BESB_083580), *Eimeria tenella* (ETH_00029095), and *Cyclospora cayetanensis* (cyc_00626), but not Piroplasms. S20 proteins contain kelch motifs, segments of approximately 50 amino acid residues that form a single four-stranded, antiparallel beta-sheet (Bork and Doolittle, 1994). Kelch motifs are widely distributed in eukaryotic and prokaryotic proteins with divergent functions. The high degree of S20 protein sequence conservation among *Plasmodium* is indicative of a possible conserved function. The sequence of H2-K^*b*^-restricted epitope VNYSFLYFL (Hafalla et al., 2013) is also relatively well maintained across *Plasmodium* species.

We next profiled *S20* expression in pre-erythrocytic stages. In good agreement with published data (Kaiser et al., 2004), we detected up-regulation of *PbS20* mRNA in midgut and salivary gland sporozoites (Supplementary Figure 1). As expected, the relative transcript abundance of *S20* was lower than the steady state levels of the *CSP* transcripts that encode the major sporozoite surface protein. Transcript levels dropped toward the end of liver stage maturation and remained low during blood infection (Supplementary Figure 1), in good agreement with the original description (Kaiser et al., 2004).

**FIGURE 2:**
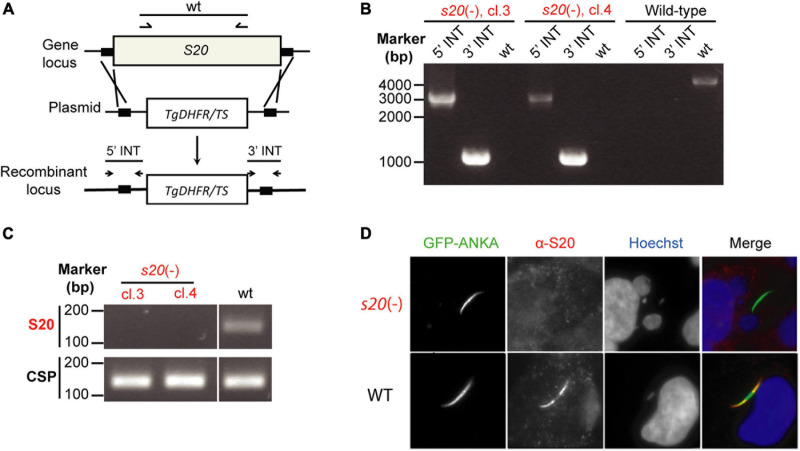
Targeted gene deletion of *P. berghei S20*. **(A)** Schematic diagram of the replacement strategy to generate *s20*(-) parasites. After linearization with *Sac*II and *Kpn*I, the replacement vector containing portions of the 5′ and 3′ flanking regions of *S20* coding sequence and the *Toxoplasma gondii dihydrofolate reductase/thymidylate synthase (Tgdhfr/ts)* selectable marker was transfected into *P. berghei* ANKA wild-type parasites. **(B)** Diagnostic PCR to confirm successful replacement of the *S20* genomic locus with the positive selection marker in *s20*(-) parasites. Shown are the results from clone 3 and 4.5′- and 3′-integration-specific primer combinations (5′ INT and 3′ INT) amplify the predicted fragment only in the recombinant locus. Wild-type-specific primer pairs (wt) do not produce a PCR fragment in the recombinant locus confirming absence of residual WT parasites. **(C)** Verification of absence of *S20* transcripts in *s20*(-) clones 3 and 4 salivary gland sporozoites by RT-PCR. Note that *s20* mRNA (top) is only detectable in WT sporozoites. *CSP* mRNA (bottom) serves as positive control. **(D)** Indirect immunofluorescent stainings of permeabilized infected hepatoma cells 1 h after *s20*(-) (top) or WT (bottom) sporozoite addition. Stainings were done with anti-GFP antibody, an anti-*Pb*S20 serum, and corresponding secondary antibodies. Shown are immune stainings, DNA stains (Hoechst, blue) and merge images.

In order to gain insights into protein expression and localization, we generated polyclonal anti-*Pb*S20 peptide sera. S20 could be detected in salivary gland sporozoites and in sporozoites that recently invaded hepatoma cells (Figure 1B). However, 24 h after invasion, S20 was no longer detectable. The S20 antisera could only recognize S20 in sporozoites that had been permeabilized, which indicates that in contrast to CSP, S20 is restricted to the sporozoite interior. The specificity of the *S20* signal in sporozoites was verified in immunofluorescence assays (IFAs) using *s20*(-) *P. berghei*, where no signal was detected (Figure 2D; see below).

### Targeted Deletion of *Plasmodium berghei S20*

We were interested in characterizing potential roles of *PbS20* for sporozoite transmission and liver infection and to explore the contribution of S20_318__–__32__6_-specific CD8^+^ T cell responses to immunity elicited by whole sporozoite immunization. However, the latter can only be tested using this strategy if *s20*(-) sporozoites induce blood infections as efficiently as wild-type (WT) sporozoites. To this end, we deleted *PbS20* by double homologous recombination to replace the open reading frame with the *Tgdhfr/ts* resistance cassette for positive selection *in vivo* (Figure 2A). Successful transfection and cloning by limiting dilution resulted in clonal *s20*(-) parasites. For this study, two clonal parasite populations, termed clones 3 and 4, were obtained after two independent transfection experiments. Genotyping by diagnostic PCR verified the desired gene deletion and absence of WT parasites from the clonal lines (Figure 2B). Additional confirmation of the absence of *S20* was obtained by RT-PCR from total RNA isolated from *s20*(-) and WT salivary gland sporozoites (Figure 2C). In contrast to *CSP* control transcripts, *S20* mRNA was only present in WT, but not in *s20*(-) sporozoites. As noted above, immunofluorescent staining using the polyclonal anti-*Pb*S20 peptide sera for WT- and *s20*(-)-infected hepatoma cells showed a loss of signal in *s20*(-), but not WT, parasites (Figure 2D). Because initial analysis revealed that both clonal parasite populations are phenotypically indistinguishable, one population (clone 4) was selected for the experiments to uncover the roles of *S20* in *Plasmodium* life cycle progression and subsequently, the H2-K^*b*^-restricted S20_318__–__326_ CD8^+^ T cell epitope in vaccine-induced protection. Swift selection of asexual *s20*(-) parasites corroborated the notion that *S20* is not required for *Plasmodium* blood infection.

### *s20*(-) Sporozoites Display Full Infectivity to Mice

Conservation of *S20* across all *Plasmodium* species might indicate critical functions during pre-erythrocytic stages. Accordingly, we monitored life cycle progression in the mosquito vector and during sporozoite transmission. Quantification of *s20*(-) midgut oocysts and salivary gland sporozoites from infected *Anopheles* mosquitoes showed no differences in comparison to WT parasites (Supplementary Table 1).

**FIGURE 3:**
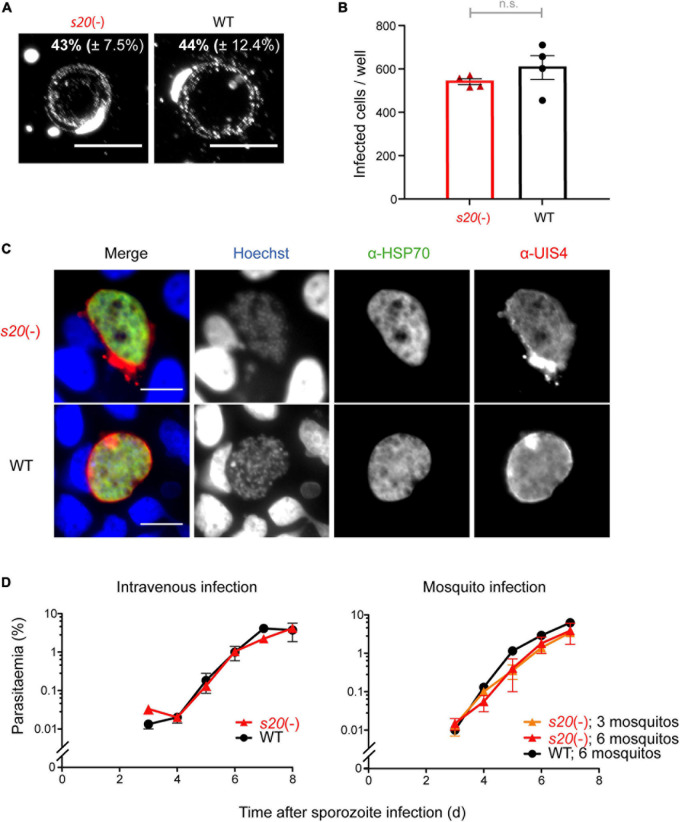
Normal life cycle progression of *s20*(-) parasites. **(A)** Fluorescence micrographs of *s20*(-) and WT sporozoites that were permitted to glide on BSA-coated glass slides. Sporozoites and trails are visualized with an anti-*Pb*CSP and anti-mouse secondary antibodies. Numbers show mean percentage (±SD) of gliding sporozoites. **(B)** Quantification of *s20*(-) and WT-infected hepatoma (Huh7) cells by fluorescence microscopy 48 h after infection. Shown are mean values (±SEM); *n* = 1; n.s., non-significant (unpaired *t*-test). **(C)** Representative fluorescence images of *s20*(-) and WT liver stages. α-HSP70 antibody (green; parasite cytoplasm), α-UIS4 antibody (red; parasitophorous vacuole), and Hoechst (blue; nucleic acid). Scale bars, 10 μm. **(D)** Blood infection after sporozoite-induced infection. B6 mice were infected by intravenous injection of 10,000 *s20*(-) or WT sporozoites (*n* = 3 each) or mosquito bites (WT, six infected mosquitoes, *n* = 1; *s20*(-), 3 or 6 infected mosquitoes, *n* = 2 each). Blood infection was monitored by daily microscopic examination of Giemsa-stained blood films. Shown are mean values (±SEM).

Salivary gland sporozoites were isolated and analyzed for their capacity to perform gliding locomotion and infect cultured hepatoma cells and mice (Figure 3). Freshly isolated *s20*(-) sporozoites displayed continuous, circular gliding locomotion on BSA-coated glass slides similar to WT parasites (Figure 3A). When deposited onto cultured Huh7 hepatoma cells, formation of liver stages was indistinguishable from WT parasites, both quantitatively and morphologically (Figures 3B,C). To rule out any defects in sporozoite infectivity *in vivo*, pre-patency, i.e., the time to detection of the first blood stage parasite, and the course of blood infection were determined by daily microscopic examination of blood films from B6 mice following intravenous injection of 10,000 *s20*(-) or WT salivary gland sporozoites, or exposure of the mice to bites of *s20*(-) or WT-infected mosquitoes (Figure 3D and Supplementary Table 2). All mice infected with either *s20*(-) or WT intravenously or by mosquito bites were blood stage-positive on day 3 after sporozoite inoculation. No differences in the course of blood infection between the two parasite groups were detectable. In good agreement, quantification of the relative liver infection load 42 h after intravenous delivery of 10,000 sporozoites by quantitative RT-PCR (qRT-PCR) revealed no difference between *s20*(-) and WT parasites (Supplementary Figure 2).

Together, *s20*(-) sporozoites displayed no apparent defects in sporozoite, liver stage or blood stage functions, permitting its use in immunization protocols.

### Effective Immunizations With Irradiated *s20*(-) Sporozoites

Intravenous immunizations with metabolically active, irradiation-attenuated sporozoites remains the benchmark for pre-clinical and clinical evaluation of experimental malaria vaccines. In humans and mice, sterile protection against sporozoite-induced infections can be routinely achieved after multiple doses of γspz (Nussenzweig et al., 1967; Hoffman et al., 2002, 2010). We initiated our study by immunizing groups of mice with three doses of 10,000 γspz. Immunized and control animals were infected by a challenge dose of 10,000 WT sporozoites 29 days after the last immunization (Table 1). All immunized animals remained blood stage negative, while the control animals became parasitaemia-positive 3 days after the challenge.

**TABLE 1:** Vaccine-induced protection against WT sporozoite challenge infections.

**Immunizations^1^**	**Protected/challenged**	**Prepatency^2^**
3 × 10,000 WT γspz	10/10 (100%)	–
3 × 10,000 *s20*(-) γspz	9/9 (100%)	–
none	0/3 (0%)	day 3

*^1^Immunizations were done by intravenous injection of irradiated (γ) sporozoites.*

*^2^Prepatency is defined as time to the first detection of a single blood stage parasite.*

**FIGURE 4:**
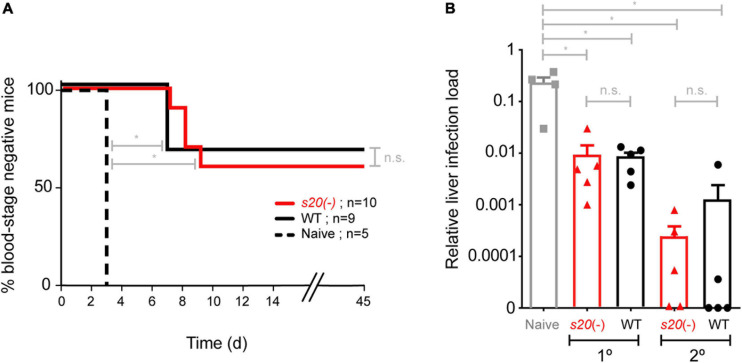
Loss of *S20* does not affect vaccine-induced protection. **(A)** Immunization and challenge protocol. Groups of B6 mice were immunized twice with 10,000 *s20*(-) (*n* = 10) or WT γ-sporozoites (*n* = 9) and challenged 35 days after the last immunization by intravenous injection of 10,000 WT sporozoites. Naïve mice (*n* = 5) served as a control. Shown is a Kaplan-Meier analysis of time to blood infection after challenge infection. From day 3 onward mice were monitored daily for blood stage parasitaemia by microscopic examination of Giemsa-stained blood films. n.s., non-significant; *p < 0.05 (Mann Whitney *U* test). **(B)** Protective efficacy as measured by qRT-PCR. *S20*(-) and WT single or double immunized were challenged with 10,000 infectious sporozoites (*n* = 5 each). Naive mice (*n* = 4) served as controls. The relative liver load was determined 42 h later after liver removal, RNA extraction and qRT-PCR with *Pb*18S rRNA primers normalized to mouse GAPDH. Shown are mean values (±SEM); *n* = 1.

In order to capture potential differences in protective efficacy offered by *s20*(-) and WT γspz, we next employed a sub-optimal immunization schedule consisting of only two doses of 10,000 *s20*(-) or WT γspz, which has been demonstrated to induce incomplete protection (Friesen and Matuschewski, 2011). Immunized mice were challenged by intravenous injection of 10,000 WT sporozoites 35 days after the last immunization (Figure 4A). Control mice became blood stage positive on day 3 after challenge inoculation. Challenge infections of *s20*(-) γspz-immunized mice resulted in blood stage parasites in 4 of 10 immunized mice with a mean pre-patent period of 8 days, whereas 3 of 9 WT γspz-immunized mice became positive for blood stage parasitaemia with a mean pre-patent period of 7 days (Figure 4A). To independently confirm the results, we performed a second immunization experiment involving single- and two dose-immunization schedule, and quantified parasite loads in the liver 48 h after challenge with 10,000 WT sporozoites by qRT-PCR (Figure 4B). In this analysis we also could not detect any differences between the relative parasite loads in livers of *s20*(-) and WT γspz-immunized mice. Together, these findings suggest that the S20_318__–__326_ reactive CD8^+^ T cell population does not contribute markedly to vaccine-induced protection.

### Lack of S20_318__–__32__6_-Specific CD8^+^ T Cells in *s20*(-) Sporozoite-Immunized Mice

**FIGURE 5:**
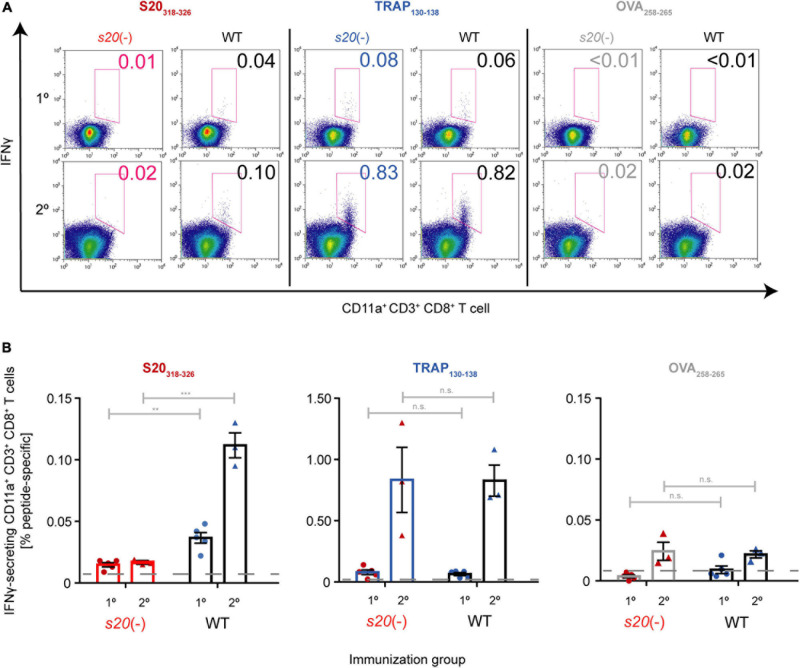
Absence of S20_318__–__326_-specific splenic CD8^+^ T cells in *s20*(-)-immunized mice. B6 mice were immunized once (1°; *n* = 5 each) or twice (2°; *n* = 3 each) with *s20*(-) (red) or WT (black) attenuated sporozoites. Peptide-specific splenic CD8^+^ T cell responses were quantified after restimulation by intracellular staining 11 or 7 days later, respectively. **(A)** Representative FACS plots showing the gating strategy to identify IFNγ-secreting CD11a^+^ CD3^+^ CD8^+^ T cells. The percentage of activated IFNγ^+^ CD8^+^ T cells among total CD8^+^ T cells after stimulation with either S20_318__–__326_ (red), TRAP_130__–__138_ (blue), or unrelated OVA_258__–__265_ (gray) peptide is indicated. **(B)** Quantification of activated, IFNγ^+^ CD11a^+^ CD8^+^ T cells among total CD8^+^ T cells after stimulation with either S20_318__–__326_ (red), TRAP_130__–__138_ (blue), or unrelated OVA_258__–__265_ (gray) peptide. Shown are mean percentage (±SEM), *n* = 1. Striped lines represent baseline responses from peptide-restimulated splenic cells of naïve mice. n.s.,non-significant; **p < 0.01; ***p < 0.001 (Unpaired *t*-test).

Finally, we wanted to unequivocally confirm that the cells in immunized mice that recognize the S20 peptide VNYSFLYLF were stimulated by this epitope in *Pb*S20. Therefore, we performed fluorescence-activated cell sorting (FACS) analysis and ELISPOT assays to detect restimulated peptide-specific splenic CD8^+^ T cells from γspz-immunized mice (Figure 5 and Supplementary Figure 3). As expected, only background signals of IFNγ responses after stimulation with S20_318__–__326_ peptide were detected in naive and *s20*(-)-immunized mice (Hafalla et al., 2013; Müller et al., 2017). In contrast, activated splenic CD8^+^ T cells from WT-immunized mice could be restimulated with S20_318__–__326_ peptide. The cell population responding to the TRAP_130__–__138_ peptide was noticeably higher, as observed in both WT- and *s20*(-)-immunized mice. As controls, we included cells from naive B6 mice (Supplementary Figure 4). Together, these results show that the S20_318__–__326_ specific responses can be attributed to the presence of *Pb*S20 in sporozoites used for immunization.

## Discussion

In this study, we deleted a previously identified CD8^+^ T cell reactive epitope together with its corresponding gene, the sporozoite-specific protein S20 (PBANKA_1429200) (Kaiser et al., 2004; Hafalla et al., 2013; Müller et al., 2017). Validating candidate antigens recognized by CD8^+^ T cells is an important aspect toward a mechanistic understanding of immune responses. As an example, previous work on identification of reactive CD8^+^ T cells in a transgenic mouse model, so called *Pb* T-1 cells, erroneously assigned the reactive peptide to PBANKA_0714500 (Lau et al., 2014) to later correct this to the ribosomal protein RPL6 (Valencia-Hernandez et al., 2020). Here, we could assign the CD8^+^ T cell-reactive, H2-K^*b*^-restricted epitope VNYSFLYFL to *P. berghei* S20. Since we also wanted to explore the cellular function of *PbS20* we employed double homologous recombination to delete the entire gene locus. We could show that *s20*(-) parasites progress normally through the *P. berghei* life cycle and induce typical exponential blood infections when animals are inoculated with *s20*(-) sporozoites. This was surprising considering the degree of conservation of S20 across the genus *Plasmodium*. The WT-like sporozoite signatures, including gliding motility, liver infectivity, and prepatency permitted us to directly compare vaccine efficacy of *s20*(-) γspz in comparison to WT γspz. Despite the absence of S20_318__–__326_-reactive, IFNγ-secreting CD8^+^ T cells we could not detect differences in γspz-induced protective efficacy in double or triple immunization protocols. An attractive hypothesis is that sporozoites express the immunogenic *S20* as a decoy to divert protective immune responses from sporozoites and developing liver stages. In support of this notion, prior studies investigating the *P. berghei* S20_318__–__32__6_ epitope uncovered a surprising lack of *in vivo* cytotoxicity of S20_318__–__32__6_-specific CD8^+^ T cells (Hafalla et al., 2013) and an inability of S20_318__–__32__6_-specific responses to confer protection in mice (Doll et al., 2016). In marked contrast, TRAP_130__–__138_-specific CD8^+^ T cells are cytotoxic, and subunit immunization strategies against *Pb*TRAP elicit protective immunity (Hafalla et al., 2013; Doll et al., 2016). The presence of a similar protein in *T. gondii*, which however lacks the CD8^+^ T cell epitope under investigation, hints at a potential alternative hypothesis. S20 could be a remnant apicomplexan sporozoite protein, which no longer exerts a central role in *Plasmodium* sporozoites. We consider this possibility less likely due to the high degree of conservation. Experimental genetics studies in coocidian parasites are warranted to determine the role of the kelch-containing proteins in these parasites and infer a potential loss of function in Haemosporidae.

Identifying immunogenic targets of protective immunity against *Plasmodium* infection remains a priority in malaria research. Early work with γspz immunizations has established that protracted, sterilizing pre-erythrocytic protection can be largely attributed to memory effector CD8^+^ T cells (Schofield et al., 1987; Weiss et al., 1988; Guebre-Xabier et al., 1999; Krzych et al., 2000; Schmidt et al., 2008). However, robust protection cannot be directly inferred from the magnitude of epitope-specific IFNγ^+^ CD8^+^ T cell populations (Hafalla et al., 2013; Kimura et al., 2013; Müller et al., 2021). For instance, heterologous prime-boost immunizations with TRAP-expressing adenovirus and Modified Vaccinia Ankara (Ad-M) generated very high levels of TRAP_130__–__138_-reactive effector CD8^+^ T cells in H2-K/D^*b*^- restricted B6 mice, but provided only partial protection against sporozoite challenge infections (Hafalla et al., 2013). On the other hand, rendering the OVA_258__–__265_ model epitope poorly immunogenic by placing it in the context of a liver stage antigen still resulted in efficient killing of parasite-infected cells by vaccination (Müller et al., 2021). In the H2-K^*d*^-restricted BALB/c model vaccine efficacy can be largely predicted by the magnitude of CD8^+^ T cells reactive to the immunodominant CSP-specific epitope (SYVPSAEQI) (Bruña-Romero et al., 2001; González-Aseguinolaza et al., 2003). Hence, in order to design subunit vaccine strategies that are as potent as the protracted sterile protection induced by γspz, many factors including specificity of CD8^+^ T cell responses and magnitude of responses have to be considered.

To date, most targets of γspz-induced CD8^+^ T cells are likely either poorly defined or remain unrecognized, and whether γspz-induced protection requires presentation of many or just a few pre-erythrocytic stage epitopes remains unclear. Previous work has focused on identifying and investigating individual CD8^+^ T cell epitopes using subunit vaccine strategies (Bruña-Romero et al., 2001; Hafalla et al., 2013; Doll et al., 2016), but the reverse approach, to test whether individual epitopes are required for sterile immunity offered by γspz, remains largely unexplored. Previous studies have demonstrated the ability to elicit sterile immunity in H-2K^*d*^-restricted BALB/c mice in the absence of CSP-specific responses (Grüner et al., 2007; Gibbins et al., 2020). The findings suggested that sterile protection does not necessarily depend on CSP-reactive CD8^+^ T cells, and indicated that inclusion of additional, non-CSP epitopes to subunit vaccine strategies is warranted. Another important stepping stone toward a better understanding of the underlying mechanisms of vaccine-induced protection is distinction of protective from non-protective epitopes by experimental genetics, as exemplified in this study.

Sterile immunity offered by γspz in the absence of *S20* clearly suggests that S20 is not a promising malaria vaccine candidate, fully supporting the findings that prime-boost immunization against S20_318__–__32__6_ epitope in mice did not elicit sterilizing immunity despite robust CD8^+^ T cell responses (Doll et al., 2016). For viral infections, compensatory CD8^+^ T cell responses were shown to be induced by subdominant epitopes when immunodominant epitope-specific effector CD8^+^ T cells are absent (Rodriguez et al., 2002; van der Most et al., 2003). Apparently, immunosuppressive effects of IFNγ secreted in large amounts by dominant CD8^+^ T cell responses are abolished, thus allowing subdominant epitope-specific precursor CD8^+^ T cells to proliferate. As a result, the subdominance is alleviated and compensatory responses are generated without suppression by IFNγ. Therefore, IFNγ plays a vital role in shaping the epitope immunodominance hierarchy, and subdominant epitopes are key players in inducing these compensatory responses in the absence of immunodominant epitopes. Together, the absence of the subdominant S20_318__–__326_ epitope in *P. berghei*, as demonstrated by inferior IFNγ responses in comparison to TRAP_130__–__138_, apparently did not result in compensatory responses generated toward other CD8^+^ T cell epitopes. Hence, maintenance of sterile protection observed in the absence of S20-specific responses was achieved by the sum of all γspz-induced responses other than S20_318__–__326_. This hypothesis can be experimentally addressed by a similar strategy as presented herein, by removal of the dominant H2-D^*b*^-restricted TRAP_130__–__138_ CD8^+^ T cell epitope.

The anti-*Pb*S20 peptide antiserum detected a cytoplasmic location of S20 in sporozoites and very early liver stages. The effect of antigen localization on protective immunity was also examined with the model antigen ovalbumin (OVA) in transgenic *P. berghei*-expressing exported or non-exported OVA (Montagna et al., 2014). *P. berghei* parasites expressing secreted OVA were found to enhance CD8^+^ T cell proliferation and MHC I presentation by infected hepatocytes during immunization, resulting in improved liver stage clearance following homologous sporozoite challenge. Importantly, large differences in immunogenicity due to antigen origin in malaria pre−erythrocytic stages are overcome by robust recognition by vaccine−induced, antigen-specific effector CD8^+^ T cells, leading to comparable high levels of protection (Müller et al., 2021). Hence, although intracellular antigens might have marginal impacts on the level of CD8^+^ T cell responses to whole sporozoite immunization, immunogenic CD8^+^ T cell epitopes derived from intracellular proteins should not be disregarded as potential candidates in subunit vaccine design.

In conclusion, our results revealed a non-vital role of *S20* in *Plasmodium* life cycle progression *in vivo* and surrogate cell culture assays, despite being a highly conserved gene across all *Plasmodium* species. Protective immunity from *s20*(-) γspz immunizations was similar to WT γspz. Our findings exemplify that malarial parasites express conserved, immunogenic proteins that are not required to establish infection in a new mammalian host but might play potential roles in diverting cellular immune responses. Molecular and immunological characterization of candidate *Plasmodium* antigens can assist in prioritizing candidates for urgently needed second-generation malaria vaccines.

## Materials and Methods

### Ethics Statement

All animal work was conducted in accordance with the German “Tierschutzgesetz in der Fassung vom 18. Mai 2006 (BGBl. I S. 1207),” which implements the directive 86/609/EEC from the European Union and the European Convention for the protection of vertebrate animals used for experimental and other scientific purposes. The ethics committee of MPI-IB and the Berlin state authorities (LAGeSo Reg# G0469/09 and G0294/15) approved the protocol.

### Parasites and Experimental Animals

*Plasmodium berghei* ANKA cl507 parasites that constitutively express GFP under the *PbEF1*α promoter were used in our experiments (Franke-Fayard et al., 2004). Six- to Eight-weeks old female NMRI and SWISS or B6 mice used in this study were either purchased from Charles River Laboratories or bred in-house. NMRI mice were used for transfection experiments, blood stage infections and transmission to *Anopheles stephensi* mosquitos. B6 mice were used for sporozoite infections and subsequent immunological studies.

### Targeted Gene Deletion and Expression of *S20*

The *P. berghei S20* gene was deleted by a double homologous recombination strategy using a standard replacement knockout plasmid (pB3D; van Dijk et al., 1995). For this aim, fragments from the 5′ and 3′ ends were amplified with primers S20SacII and S20NotI, and with the primer pair S20HindIII and S20KpnI using PCR. Subsequently, the 5′ PCR fragment was double digested overnight at 37°C with the restriction enzymes *Sac*II and *Not*I, while the restriction enzymes *Hind*III and *Kpn*I were used for the 3′ fragment digestion. 10 μg plasmid was linearized with *Sac*II and *Kpn*I for transfection. Transfection experiments were carried out twice, as previously described (Janse et al., 2006), to obtain two independent clones for phenotypical analysis; clone 3 and 4. Successful integration was tested on genomic DNA by conventional PCR using the primers Test1S20fw and JFUTRrv for 5′ integration, and TgPro and Test2S20rv for 3′ integration. Absence of WT parasites in the clonal population was verified using Test1S20fw and Test2S20rv primers. The PCR was carried out as follows: Initial denaturation for 3 min at 94°C, followed by 35 cycles of 30 s denaturation at 94°C, annealing for 45 s at 55°C, and extension at 60°C for 2 min 30 s. A final extension was carried out for 10 min at 60°C. For studying the expression of *S20* during the *P. berghei* life cycle, amplification was performed by qPCR with the primers qS20fw and qS20rv on cDNA of midgut sporozoites, salivary gland sporozoites, early liver stages, late liver stages, mixed blood stages and gametocytes obtained from *P. berghei* ANKA cl507 parasites, which constitutively express GFP under the *PbEF1*α promoter (Janse et al., 2006). Expression levels were normalized to the *GFP* transcript levels determined by primers qGFPfw and qGFPrv.

### Salivary Gland Sporozoite Isolation

Wild-type GFP-expressing *P. berghei* ANKA and *P. berghei s20*(-) strains were maintained by continuous cycling between rodent hosts (B6 or NMRI and SWISS mice) and female *A. stephensi* mosquito vectors (Vanderberg, 1975). Mosquitoes were kept at 28°C (non-infected) or 20°C (infected) at 80% humidity. After 10–14 days, midguts were isolated and oocyst development analyzed by fluorescent microscopy. Salivary gland sporozoites were isolated from mosquitos in DMEM medium containing 10% fetal calf serum (FCS).

### Polyclonal Anti-S20 Antibody Generation

Polyclonal antibodies against S20 were raised in rabbits immunized with synthetic peptides (MSDISDFSDIDDFSE+C and C+TFTSKKLTENPGKRAY) (Eurogentec, Seraing, Belgium).

### Immunofluorescence Staining of Salivary Gland Sporozoites

The gliding motility of salivary gland sporozoites was evaluated by IFA. 5,000 WT or *s20*(-) salivary gland sporozoites suspended in 3% BSA-RPMI were added into each ring of a Medco glass slide pre-coated with 3% BSA-RPMI. The sporozoites were incubated for 45 min at 37°C, during which the parasites glide and shed surface proteins into the extracellular environment. To visualize gliding motility, sporozoites and trails of shed proteins were stained with mouse anti-sporozoite surface antibody, followed by anti-mouse Alexa Fluor 488-coupled antibody (Invitrogen, Carlsbad, CA, United States) and the nuclei stain Hoechst 33342 (Invitrogen, Carlsbad, CA, United States) Slides were mounted with Fluoromount-G (SouthernBiotech) prior to analysis by fluorescence microscopy using Zeiss Axio Imager Z2. To detect S20 by immunofluorescence, sporozoites were fixed in 3% PFA and permeabilized as indicated with 0.3% Triton X-100 prior to staining with custom-made anti-S20 peptide antiserum or monoclonal anti-CSP (3D11) antibody (Yoshida et al., 1980), and Hoechst 33342 (Invitrogen, Carlsbad, CA, United States).

### Hepatoma Cell Infection *in vitro* and Immunofluorescence Staining

Nunc Lab-Tek II 8-well Chamber Slides (Thermo Fisher Scientific, New York, NY, United States) were seeded with 30,000 Huh7 cells per well 24 h before infection. 10,000 WT or *s20*(-) salivary gland sporozoites suspended in 100 μl DMEM-complete were added and allowed to settle for 1 h at RT. *P. berghei* S20 was immunostained to investigate the expression of *S20* during liver stage development. Here, infected hepatoma cells were incubated at 37°C for 1 and 3 h after infection prior to fixation with 4% paraformaldehyde and staining with monoclonal anti-HSP70 antibody (Tsuji et al., 1994), anti-S20 peptide antiserum, and Hoechst 33342 (Invitrogen, Carlsbad, CA, United States). Phenotypic analysis of parasite liver stage development was monitored by IFA in Huh7 cells 48 h post-infection at 37°C. The infected cells were permeabilized with 0.2% Triton-X 100 (Roth, Karlsruhe, Germany) and analyzed 48 h later after fixation with 4% PFA by staining with mouse anti-HSP70 antibody (Tsuji et al., 1994) followed by goat anti-mouse Alexa Fluor 488 antibody (Invitrogen, Carlsbad, CA, United States) and Hoechst 33342 (Invitrogen, Carlsbad, CA, United States). Slides were mounted with Fluoromount-G (SouthernBiotech) and imaged by fluorescence microscopy (Zeiss Axio Imager Z2). At least 20 intracellular parasites per sample were visualized.

### *s20*(-) Parasite Infectivity *in vivo*

To determine the infectivity of sporozoites, B6 mice were infected intravenously with either 10,000 WT or *s20*(-) salivary gland sporozoites suspended in 100 μl PBS per mouse. Simultaneously, another group of B6 mice were infected through direct bites from WT or *s20*(-)-infected *A. stephensi* mosquitos for 20 min. In order to confirm the number of mosquitos that received a blood meal, the mosquitos were paralyzed briefly at −20°C and observed for blood in the abdomen under a compound microscope. The pre-patent period was determined by daily microscopic examination of Giemsa-stained blood smears.

### Quantification of Parasite Development *in vivo*

To rule out a defect of *s20*(-) parasites during liver stage development and to determine protective outcome of immunized mice against homologous sporozoite challenge, *P. berghei* 18S rRNA level during liver stage infection was quantified. Naïve B6 mice were inoculated with either 10,000 WT or *s20*(-) sporozoites intravenously for phenotypic characterization, and in the sterile protection experiment, female B6 mice were challenged with 10,000 WT sporozoites 35 days after the last immunization. 42 h after infection, the mice were sacrificed and livers were removed to isolate total RNA using Qiagen RNeasy kit, followed by reverse transcription of the RNA using RETROscript kit. Quantitative Real-Time PCR was performed on cDNA samples using Pb18S ribosomal subunit primer pairs (Gene ID: 160641; sense and antisense primers), mouse GAPDH primer pairs (Gene ID: 281199965; sense primer and antisense primers). Relative liver infection loads were then determined using the ΔC_*t*_ method as previously described (Friesen et al., 2010).

### Immunization With Attenuated WT and *s20*(-) Sporozoites

Salivary gland sporozoites were attenuated either by a γ-irradiation dose of 12 × 10^4^ cGy, or with azithromycin (AZ) cover (4.8 mg in 200 μl of sodium chloride solution per mouse) (Friesen et al., 2010). For challenge experiments, B6 mice were immunized with two and three doses of WT and *s20*(-) γspz. Immunizations in the two-dose regimen with 10,000 sporozoites were carried out on day 0 and 7. Challenge with 10,000 sporozoites was on day 42, including an addition of 5 age-matched naïve B6 mice. The priming dose of the three-dose regimen consisted of 15,000 sporozoites, while the subsequent two boosts comprised of 10,000 sporozoites. The immunization doses were given at days 0, 35, and 55, followed by a challenge with 10,000 WT sporozoites on day 84, including three naïve animals. All mice were checked for parasitemia by microscopical examination of Giemsa-stained blood smears starting at day 3 after challenge inoculation until day 14. Those animals that remained parasitemia-free were continuously checked for parasitemia up till 45 days after the challenge.

### Quantification of S20_318__–__326_-Specific Splenic CD8^+^ T Cells in Immunized Mice

Each B6 mouse in immunological studies was immunized intravenously with attenuated sporozoites resuspended in 1× PBS *via* the lateral tail vein. FACS after restimulation and staining was done to determine the absence of S20_318__–__326_-specific splenic CD8^+^ T cell responses. B6 mice were immunized with either a single-dose of 15,000 AZ-attenuated sporozoites, or two-dose regimen of γspz followed by AZ-attenuated sporozoites carried out on day 0 and 7, respectively, where each dose consisted of 10,000 sporozoites. B6 mice used in enzyme-linked immune absorbent spot (ELISPOT) experiments were immunized twice with irradiated *s20*(-) or WT irradiated sporozoites at days 0 and 7. Another two groups were immunized in parallel at day 7. Sixteen days after the last immunization, animals were challenged with 10,000 sporozoites, including five naïve mice. 42 h after challenge mouse spleens were used to extract CD8^+^ T cells and analyzed for their capacity to induce IFNγ responses specific for S20_318__–__326_ and TRAP_130__–__138_-specific CD8^+^ epitopes.

The peptides VNYSFLYLF (S20_318__–__326_), SALLNVDNL (TRAP_130__–__138_) (Hafalla et al., 2013), and SIINFEKL (OVA_258__–__265_) were synthesized by Peptides & Elephants (Potsdam, Germany), and reconstituted in DMSO/water (1:1) at a concentration of 1 mM. Female B6 mice that received a single-dose or prime-boost sporozoite immunization were dissected 11 and 7 days after last immunization, respectively, along with naïve control animals, to obtain splenic lymphocyte cell suspension using a cell strainer. Red blood cell lysis was then carried out with BD Pharm Lyse^TM^ lysing buffer (Becton, Dickinson and Company, United States) as described in the manufacturer’s protocol. In order to quantify sporozoite-induced CD8^+^ T cells, the lymphocytes were restimulated with peptides for 5–6 h in the presence of Brefeldin A (Invitrogen, Carlsbad, CA, United States), followed by extracellular and intracellular staining procedures specific for CD3, CD11a, CD8a, and IFNγ using the following anti-mouse antibodies; CD3e PE-Cy7 (eBioscience, Inc., San Diego, CA, United States), CD8a PerCP Cy5.5 (eBioscience, Inc., San Diego, CA, United States), CD11a e450 (Invitrogen, United States), and IFNγ-APC (eBioscience, Inc., San Diego, CA, United States). The cells were analyzed using a BD LSR Fortessa flow cytometer (Becton, Dickinson and Company, United States) and data analysis was performed on FLOWJO software (Becton, Dickinson and Company, United States). Target CD8^+^ T cells were identified from the gated lymphocyte population by the removal of doublets followed by the exclusion of cell debris, artifacts, and other non-target cells (Supplementary Figure 4B).

To support the findings from FACS analysis, ELISPOTs were also carried out on splenic CD8^+^ T cells from immunized female B6 mice (see above) using the same VNYSFLYLF (S20_318__–__326_) and SALLNVDNL (TRAP_130__–__138_) (Hafalla et al., 2013) peptides for restimulation. Sterile high protein binding Immobilon-P MultiScreen 96-Well Plate (Millipore, Germany) with 0.45 μm pore size were used as ELISPOT plates. Prior to plating of the cells, plates were pre-wet for 1 min with 35% EtOH, washed with sterile water and incubated overnight with 75 μl of rat anti-mouse IFNγ antibody (AN-18; rat IgG1; eBioscience) at a concentration of 8 μg/ml in sterile PBS at 4°C. After washing with sterile PBS, plates were blocked with DMEM containing 10% FCS and Penicillin/Streptomycin for 2 h at 37°C. Splenic cell suspensions from immunized and control animals were prepared using a cell strainer, and red blood cells were lysed with BD Pharm Lyse lysing buffer as described in the manufacturer’s protocol. 10^8^ cells were used for isolation of CD8^+^ cells by positive selection using CD8a (Ly-2) MicroBeads (Miltenyi, Germany) following the manufacturer’s instructions. 200,000 CD8^+^ cells and the same number of antigen-presenting cells were used per well. Naïve spleen cells were incubated with 2 μM peptide for 2 h at 37°C. Afterward, antigen-presenting cells were γ-irradiated for 28 min and washed twice before co-incubation for at least 20 h with CD8^+^ T cells at 37°C. Plates were washed three times with 1× PBS and three times with 1× PBS containing 0.05% Tween 20 (PBST20), and were subsequently incubated with 100 μl PBST20 containing 0.5% FCS with 1 μg/ml of the biotinylated detection antibody anti-mouse IFNγ mAb R4-6A2 (Mabtech, Germany) overnight at 4°C. On the following day, plates were washed four times in PBST20 and incubated further for 1 h at room temperature with streptavidin-conjugated alkaline phosphatase (BD Pharmingen, Germany) in PBST20 containing 1% FCS. After washing four times with PBST20 and 1× PBS, ELISPOT plates were developed using the Bio-Rad AP color development kit until spots were visible, which were then counted using a binocular.

### Statistics

Statistical analysis (see Figure Legends) were performed using Prism software (GraphPad Software Inc., United States).

## Data Availability Statement

The original contributions presented in the study are included in the article/Supplementary Material, further inquiries can be directed to the corresponding author/s.

## Ethics Statement

The animal study was reviewed and approved by the Ethics Committee of MPI-IB and the Berlin state authorities (LAGeSo Reg# G0469/09 and G0294/15) approved the protocol.

## Author Contributions

KMa, JF, CH, JH, and KMü designed the experiments. CH and JF performed the experiments and analyzed the data. AI performed and analyzed the sporozoite IFA. DS generated knockout parasite lines. KMa, CH, and JF wrote the manuscript. All authors commented and revised the manuscript.

## Conflict of Interest

The authors declare that the research was conducted in the absence of any commercial or financial relationships that could be construed as a potential conflict of interest.

## Publisher’s Note

All claims expressed in this article are solely those of the authors and do not necessarily represent those of their affiliated organizations, or those of the publisher, the editors and the reviewers. Any product that may be evaluated in this article, or claim that may be made by its manufacturer, is not guaranteed or endorsed by the publisher.
