# Supplementary material for: Conservation of S20 as an Ineffective and Disposable IFNγ-Inducing Determinant of Plasmodium Sporozoites Indicates Diversion of Cellular Immunity
Source: Front Microbiol. 2021 Aug 6;12:703804. doi: 10.3389/fmicb.2021.703804 (PMC8377727; doi:10.3389/fmicb.2021.703804)
Supplement: Supplementary file 1 [file Data_Sheet_1.PDF]

Supplementary Material

Conservation of S20 as an ineffective and disposable IFNγ-inducing determinant of *Plasmodium* sporozoites indicates diversion of cellular immunity

Calvin Hon, Johannes Friesen, Alyssa Ingmundson, Diana Scheppan, Julius C.R. Hafalla, Katja Müller, and Kai Matuschewski

Contents:

- **Figures S1 - S4**
- **Tables S1 – S3**

Hon *et al.*, Figure S1

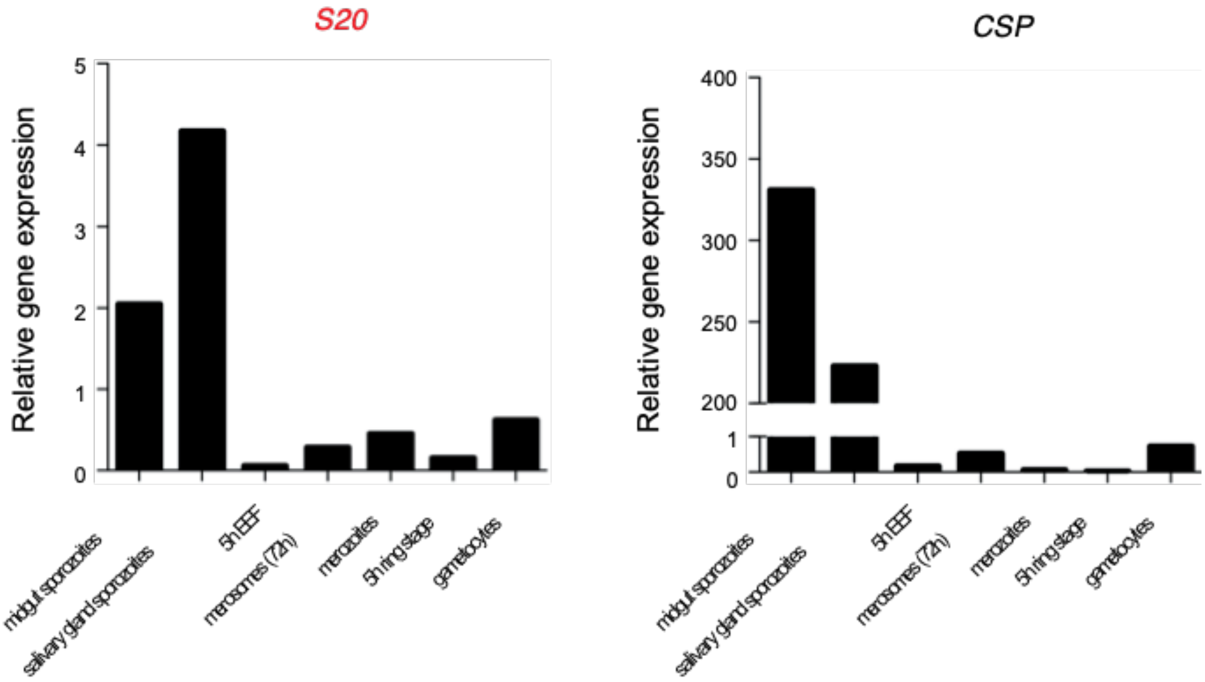

Supplementary Figure 1. S20 transcripts were mainly detected in midgut and salivary gland sporozoites. Transcript levels of *S20* and *CSP* in *P. berghei* were assessed in midgut and salivary gland sporozoites, 5 h *in vitro* infected hepatoma cells, 72 h merosomes, merozoites, 5h ring stage parasites, and gametocytes.

Hon *et al.*, Figure S2

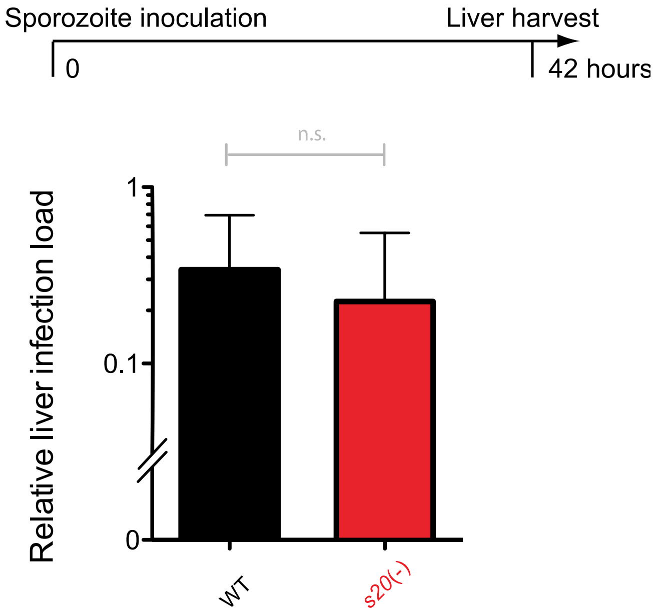

Supplementary Figure 2. *In vivo* quantification of *s20*(-) liver stages by quantitative Real Time-PCR. To confirm the absence of developmental anomalies in *s20*(-) parasites during liver stage development, C57BL/6 mice (*n*=5 per group) were injected with 10,000 WT (black) or *s20(-)* (red) sporozoites. 42 hours later, the mice were sacrificed and livers were removed for RNA extraction to determine the mean relative liver infection load (±SEM) by quantitative Real-Time PCR. n.s., non-significant (Unpaired *t*-test)).

Hon *et al.*, Figure S3

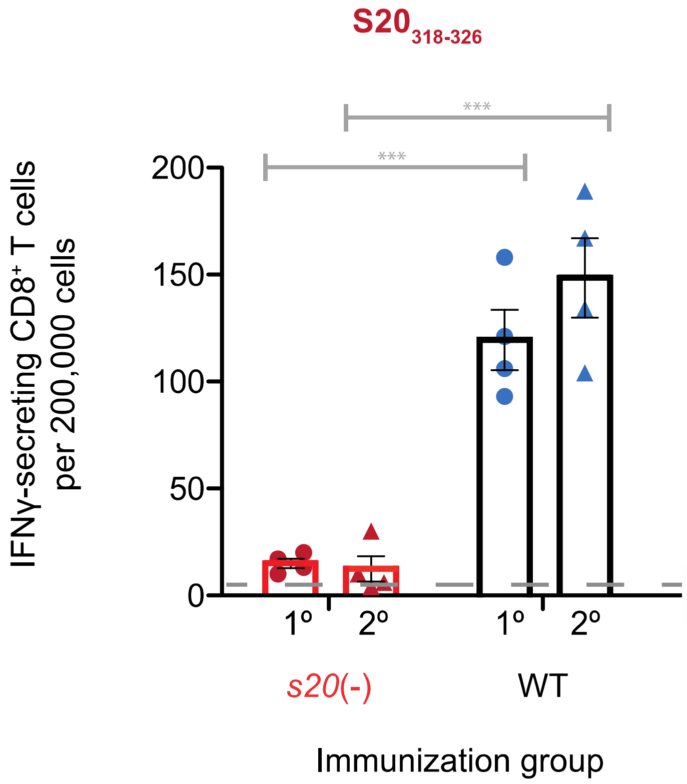

Supplementary Figure 3. ELISPOT confirmed absence of S20$_{318\text{-}326}$ epitope in *s20*(-) *P. berghei.* Splenic CD8^{+} T cell responses in B6 mice immunized with *s20*(-) and WT γspz, as well as naïve control mice (*n*=4 per immunization group) were analyzed for their capacity to induce IFNγ responses specific for CD8^{+} T cell epitopes S20$_{318\text{-}326}$, TRAP$_{130\text{-}138}$, and OVA$_{258\text{-}265}$ after peptide restimulation. Bar graph shows the mean number (±SEM) of visible spots. *n*=1.

Hon *et al.*, Figure S4

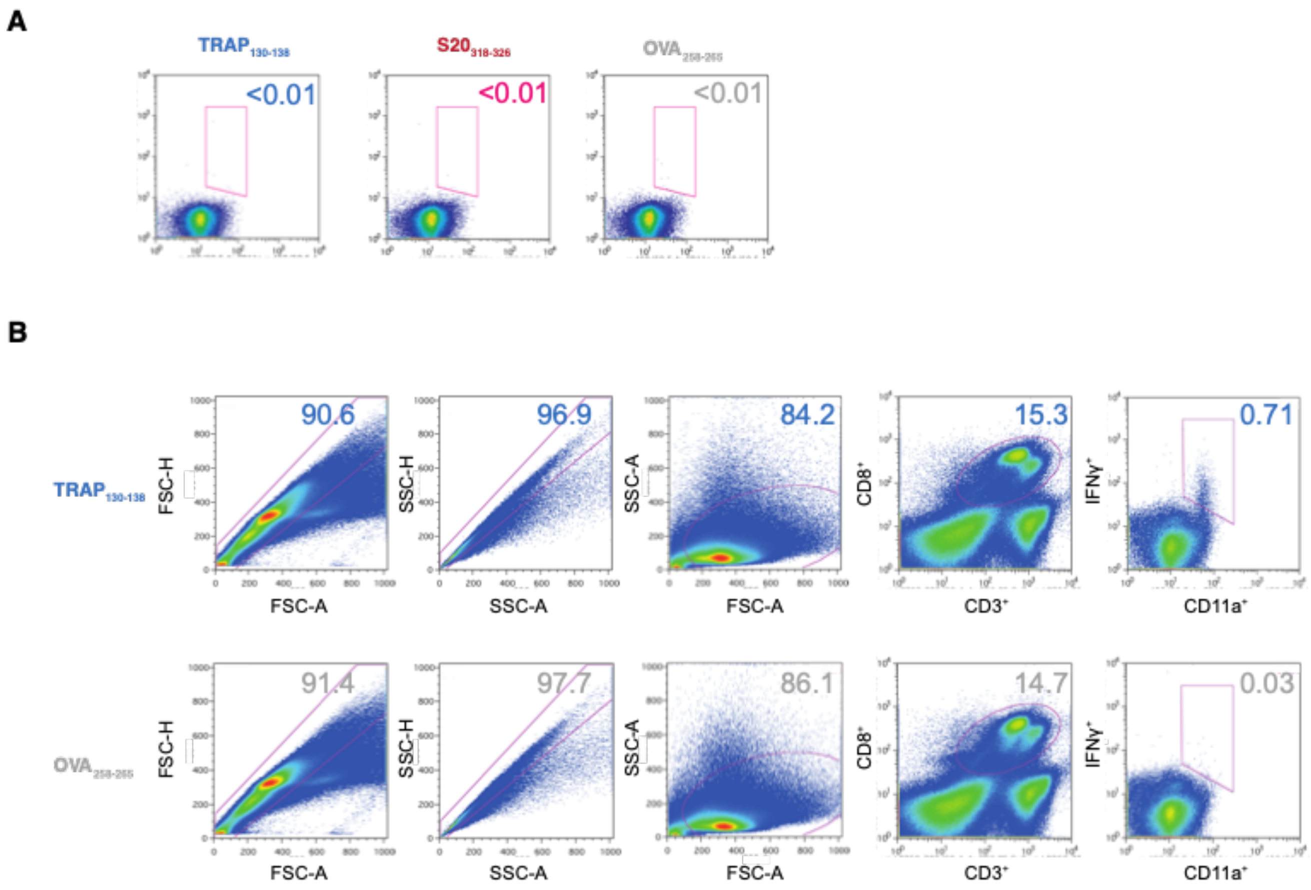

Supplementary Figure 4. Absence of peptide-specific splenic CD8+ T cell responses in naïve B6 mice and gating strategy for splenic lymphocytes illustrating and a

(A) IFNγ-secreting CD8+ T cell responses of a naïve non-immunized B6 mouse (*n*=1) were quantified in comparison to *s20*(-)- and WT-immunized mice by intracellular staining (see Figure 5).

(B) Representative FACS plots showing the gating strategy for identifying IFNγ-secreting CD11a+ CD8+ T cell population, including the mean percentage of activated IFNγ+ CD8+ T cells among total splenic lymphocyte population.

Supplementary Table 1. Midgut oocysts and salivary gland sporozoites from *s20*(-) and WT-infected *Anopheles* mosquitoes

**Parasite**	**% infectivity (d10 midgut oocysts)**	**# salivary gland sporozoites per mosquito**[1]
WT	64% (± 26%)	11,300 (± 5,400)
s20(-)	75% (± 21%)	15,300 (± 2,400)

[1]An average of 18 mosquitos were dissected for salivary gland sporozoites.

Supplementary Table 2. Prepatency of *s20*(-) and WT sporozoite infections

	**Prepatency (dpi)**	
**Route of infection**	**WT**	***s20*(-)**
i.v. (sporozoite)[1]	3.0	3.0
Mosquito bite (x3)	3.0	3.0
Mosquito bite (x6)	3.0	3.0

[1] intravenous injection of 10,000 sporozoites per mouse

Supplementary Table 3: Oligonucleotides used in this study

Name	Restriction site	Sequence	Purpose
**Oligonucleotides used for gene deletion constructs and for integration-specific PCRs:**			
S20SacII	*Sac*II	TCCCCGCGGCATCGGAATATTCCTTTCTATA	Knockout *S20*
S20NotI	*Not*I	ATAAGAATGCGGCCGCTGTAAATTTTTTATATATTTAT	Knockout *S20*
S20HindIII	*Hind*III	GGGAAGCTTTTCTGCTTAGCTTCTATAAAGC	Knockout *S20*
S20KpnI	*Kpn*I	GGGGTACCGAAGGATTTTTCAACATCAGAC	Knockout *S20*
Test1S20fw	-	TTTTATTTTATTATTTTTTTTTTCGAACTATATT	Integration *S20*
Test2S20rv	-	AATGGGGAAATATCA ATATTCA	Integration *S20*
JFUTRrv	-	AATTCCGGTGTGAAATACCGCACAGA	Integration plasmid
TgPro	-	CGCATTATAT GAGTTCATTTTACACAATCC	Integration plasmid
**Oligonucleotides used for qPCR:**			
qS20fw	-	GTGAAGATAAAGGATTACTTATGGA	qPCR *S20*
qS20rv	-	CATCAGTTCGCTGTTGTTGGTC	qPCR *S20*
qGFPfw	-	GATGGAAGCGTTCAACTAGCAGACC	qPCR *GFP*
qGFPrv	-	AGCTGTTACAAACTCAAGAAGGACC	qPCR *GFP*
18S sense	-	AAGCATTAAATAAAGCGAATACATCCTTAC	qPCR *18S rRNA*
18S antisense	-	GGAGATTGGTTTTGACGTTTATGTG	qPCR *18S rRNA*
mGAPDH sen	-	CGTCCCGTAGACAAAATGGT	qPCR *mGAPDH*
mGAPDH as	-	TTGATGGCAACAATCTCCAC	qPCR *mGAPDH*
